# Estrategia de tamizaje para la selección de epítopos de ***Leishmania*** spp. en humanos por medio del análisis de interacciones moleculares: un acercamiento desde la bioinformática

**DOI:** 10.7705/biomedica.8036

**Published:** 2026-08-20

**Authors:** Magda Melissa Flórez, Julián Rodríguez, Francy Elaine Torres

**Affiliations:** 1 Grupo de Investigación, Facultad de Medicina, Universidad Cooperativa de Colombia, Santa Marta, Colombia Universidad Cooperativa de Colombia Universidad Cooperativa de Colombia Santa Marta Colombia; 2 Centro de Investigación en Ciencias de la Vida, Universidad Simón Bolívar, Barranquilla, Colombia Universidad Simón Bolívar Universidad Simón Bolívar Barranquilla Colombia

**Keywords:** Leishmania, leishmaniasis, epítopos de linfocito T, antígenos HLA-DR, biología computacional, Leishmania, leishmaniasis, epitopes, T-lymphocyte, HLA-DR antigens, computational biology

## Abstract

**Introducción.:**

El parásito *Leishmania* es fagocitado por los macrófagos y se aloja en el fagolisosoma; por consiguiente, se necesita la activación por los linfocitos CD4-Th1 para controlar la infección. La presentación antigénica es crítica para dicha activación al presentar secuencias cortas del parásito en el bolsillo de las moléculas del complejo mayor de histocompatibilidad (CMH) de clase II.

**Objetivo.:**

Identificar las secuencias lineales de proteínas inmunogénicas de *Leishmania (Viannia)* spp., mediante herramientas bioinformáticas frente a moléculas humanas del CMH de clase II.

**Materiales y métodos.:**

Se tomaron secuencias lineales de las proteínas GP63, HSP70 y PEPCK de *Leishmania panamensis* y se predijo la unión de secuencias de 15 aminoácidos frente a diversos HLA-DRß*04 por medio de herramientas de predicción. Se confirmó que las regiones están muy conservadas entre las especies del parásito, con nula o poca analogía con las proteínas humanas. Finalmente, se analizaron las interacciones de los péptidos con mejores resultados por acoplamiento molecular frente al HLA-DRß*04:01.

**Resultados.:**

En la primera fase del estudio, se identificaron seis secuencias con gran potencial de ser epítopos T naturales de *Leishmania* spp. Las secuencias GP63_267-282_, GP63_282-297_ y PEPCK_535-550_ presentaron múltiples interacciones y bajas energías de interacción frente al bolsillo del HLA-DRß*04:01 y orientación de N-terminal a C-terminal.

**Conclusiones.:**

Se identificaron tres secuencias como posibles epítopos Th1 de *Leishmania* spp.; sin embargo, es necesaria la validación *in vitro* para confirmar estos hallazgos.

## Abstract (en)

**Introduction.:**

The *Leishmania* parasite is phagocytized by macrophages and stored in the parasitophorous vacuole; therefore, the activation of Th1-CD4^+^ lymphocytes is essential to control the infection. Consequently, antigen presentation is critical for such activation, occurring through the binding of short parasite sequences to the MHC class II pocket.

**Objective.:**

To identify linear sequences from immunogenic proteins of *Leishmania (Viannia)* spp. through bioinformatic analysis of human MHC class II molecules.

**Materials and methods.:**

Linear sequences of GP63, HSP70, and PEPCK were obtained from *Leishmania panamensis,* and the binding of 15-mer peptides to various HLA-DRß*04 alleles was predicted. Highly conserved sequences among parasite species with null or low analogy to human proteins were selected. Finally, molecular docking was performed against the HLA-DRß*04:01 pocket.

**Results.:**

Six sequences were initially selected as potential natural T-cell epitopes. Among these, GP63_267-282_, GP63_282-297_, and PEPCK_535-550_ demonstrated multiple interactions with the HLA-DR: 04:01 pocket and a consistent N-terminus to C-terminus orientation. Conclusion. Three sequences were identified as potential *Leishmania* Th1 epitopes; however, *in vitro* validation is necessary to confirm these findings.

El género *Leishmania* está conformado por parásitos protozoarios intracelulares distribuidos en las zonas tropicales y subtropicales del planeta. Este género tiene gran relevancia clínica mundial por su capacidad para infectar seres humanos al transmitirse por la picadura de insectos hematófagos del género *Lutzomyia* en el Nuevo Mundo. Mundialmente, se reportan entre 0,9 y 1,6 millones de casos anuales en 98 países afectados ^1^, y la presentación cutánea es la forma clínica más prevalente.

En el continente americano, se presentan cerca de 64 000 casos anuales; Brasil, Perú y Colombia son los países que reportan el mayor número de casos. En Colombia, se reportó una media anual de 7481 casos en la última década y la leishmaniasis cutánea fue la forma predominante con más del 98 % de los casos, causada principalmente por las especies *Leishmania panamensis, L. braziliensis* y *L. guyanesis* fueron las más prevalentes ^1^^,^^2^.

A nivel inmunológico, para una eficiente eliminación del parásito se requiere la participación de los linfocitos T *helper* 1 (Th1) para que activen los macrófagos (célula blanco del parásito) por medio del IFNy. Por lo tanto, es primordial la correcta activación de los linfocitos por parte de las células presentadoras de antígeno. Estas células fagocitan al parásito, procesan sus proteínas en el fagolisosoma y presentan fragmentos (o péptidos) de estas a los linfocitos T CD4+ por medio de los antígenos leucocitarios humanos *(Human Leukocyte Antigens,* HLA) de clase II en la superficie de su membrana que, junto con el perfil de citocinas del microambiente, inducen la activación y diferenciación celular de los linfocitos Th ^3^.

Este proceso es de una complejidad considerable y está determinado, no solo por la naturaleza de las proteínas del parásito y los péptidos producto de su degradación, sino también, por las características de los HLA presentes en el huésped. La afinidad entre estos dos últimos es la que determina las secuencias que se presentan en los linfocitos T y, por consiguiente, el curso de la respuesta inmunológica y el desarrollo de inmunidad ^4^^,^^5^. Por otro lado, los HLA son muy variables entre las poblaciones humanas, e incluso entre individuos, lo que implica que el mismo péptido podría no ser inmunogénico en dos poblaciones diferentes.

Dada la gran cantidad de variables que intervienen en las dinámicas inmunológicas entre el huésped y el agente patógeno, resulta difícil cubrir cada una de las posibilidades en los modelos experimentales *in vitro* e *in vivo.* Afortunadamente, con el auge del aprendizaje automático *(machine learning)* de las últimas décadas, se han creado herramientas bioinformáticas que posibilitan el estudio y la predicción del comportamiento de sistemas biológicos complejos. Estas herramientas permiten obtener estos datos entre moléculas concretas, lo cual posibilita realizar un tamizaje para seleccionar unos pocos del gran número de candidatos que posteriormente pueden ser evaluados *in vitro,* optimizando así tiempos, costos y mano de obra en las fases preclínicas del desarrollo de vacunas ^6^.

Las proteínas que son ideales para la identificación de epítopos lineales son aquellas que están muy conservadas en las especies de *Leishmania* y que no tienen regiones idénticas o análogas en los humanos. La glicoproteína de membrana GP63, o leishmaniolisina, y la proteína de choque térmico 70 (HSP70), entre otras, han sido identificadas como moléculas antigénicas mayores en *Leishmania* spp. ^7^^-^^14^.

Teniendo en cuenta lo anterior, la presente investigación tuvo como objetivo identificar *in silico* péptidos con potencial inmunogénico de las proteínas GP63, HSP70 y PEPCK de las especies de *Leishmania* circulantes en Colombia, y su afinidad por HLA-DR, comunes en la población colombiana.

## Materiales y métodos

### Selección y obtención de datos de proteínas

Se hizo una búsqueda de informes de proteínas en revistas indexadas en ScienceDirect, Wiley, Nature, Scopus y SpringerLink, y se seleccionaron aquellas que cumplieran con los siguientes criterios:

1. la proteína ha sido caracterizada a nivel de secuencia en el genoma de *Leishmania panamenisis, L. braziliensis* o *L. guyanensis;*
2. la proteína se expresa de forma constitutiva en las dos formas del parásito;
3. la proteína tiene actividad inmunogénica comprobada, y
4. la proteína tiene poca similitud con homólogos humanos.

### Análisis in silico

La secuencia completa de cada proteína se obtuvo en formato FASTA de la base de datos del *National Center for Biotechnology Information* (NCBI) de la *National Library of Medicine.*

Las proteínas de *L. panamensis* seleccionadas fueron:

1. la glicoproteína de membrana (GP63) o leishmaniolisina (AAB97896.1);
2. la proteína de choque térmico 70 (HSP70) (Heaí *Shock Protein* 70) (XP_010700632.1), y
3. la fosfoenolpiruvato carboxicinasa (PEPCK, *phosphoenolpyruvate carboxykinase)* (XP_010696875.1).

A partir de la secuencia lineal de aminoácidos de cada proteína, se realizaron alineamientos múltiples de la secuencia de *L. panamensis* frente a *L. braziliensis, L. guyanensis* y *Homo sapiens* por medio de las herramientas BLAST *(Basic Local Alignment Search Tool)* y MUSCLE *(Multiple sequence comparison by log-expectation)*^15^, para confirmar la analogía entre las especies de *Leishmania* y la escasa analogía con su respectivo homólogo humano.

La representación de la proteína se obtuvo de la predicción de la estructura tridimensional generada por Alphafold ^16^^,^^17^. La edición de las figuras fue hecha con el *software* Pymol ^18^.

### Predicción de unión de péptidos y HLA-DR

Las secuencias lineales de cada proteína se analizaron con dos herramientas diferentes de predicción de epítopos T: NetMHCIIpan ^19^ y MixMHC2pred ^20^, validadas por su capacidad para predecir la interacción entre péptidos y alelos del complejo mayor de histocompatibilidad (CMH). Las dos herramientas hicieron predicciones de unión de secuencias de 15 aminoácidos a lo largo de toda la proteína frente a los siguientes 11 alelos de HLA-DR04: DRB1_04:01, DRB1_04:02, DRB1_04:03, DRB1_04:04, DRB1_04:05, DRB1_04:06, DRB1_04:07, DRB1_04:08, DRB1_04:10, DRB1_04:11 y DRB1_04:17 ^21^. Este grupo de alelos es más frecuente en la población colombiana ^22^.

### Preparación del receptor (HLA-DR4) y de los péptidos derivados de las proteínas GP63 y PEPCK

La proteína HLA-DR4 (4mdj) se descargó del *Protein Data Bank* (PDB) (https://www.rcsb.org/structure/4MDJ) y se preparó en Atuodock vina y DStudio visualizer (BIOVIA, https://discover.3ds.com/discovery-studio-visualizer-download). Para esto, al receptor se le removieron los ligandos presentes y las moléculas de agua, y se le adicionaron fuerzas de Kollman y átomos polares. Además, la proteína se revisó manualmente, para determinar el bolsillo de unión y la estructura secundaria. El archivo fue depositado en formato PDB para su posterior uso en el acoplamiento molecular.

Los péptidos generados por las herramientas mencionadas se modelaron por medio de la herramienta PEP-FOLD, versión 4.5 (https://bioserv.rpbs.univ-paris-diderot.fr/services/PEP-FOLD/) y los archivos generados se almacenaron en formatos PDB para ser usados posteriormente. Se seleccionaron los mejores modelos, basados en el análisis del diagrama de Ramachandran y en su índice de toxicidad (https://webs.iiitd.edu.in/raghava/toxinpred/algo.php).

### Acoplamiento molecular HLA-DR4: péptidos derivados de proteínas

Se llevó a cabo el acoplamiento molecular, por medio de la herramienta MDOckPEP (https://zougrouptoolkit.missouri.edu/mdockpep/index.html) ^23^, en la cual se usó como receptor la proteína HLA-DR4 preparada previamente y las secuencias peptídicas generadas. Se seleccionaron los mejores modelos de interacción (kj/mol) por cada péptido evaluado y se identificaron, por medio de DStudio visualizer, las propiedades de unión -número de interacciones favorables y no favorables, totalidad de puentes de hidrógeno, interacciones hidrofóbicas y puentes de sal-, que le otorgan al complejo la estabilidad (térmica, pH y electrostáticas).

## Resultados

El proceso inicial de tamizaje constó de tres fases. Primero, la identificación de secuencias como posibles epítopos T de *Leishmania* spp. mediante la evaluación de la unión de cada secuencia a los diferentes alelos de HLA-DRß*04 comunes en la población colombiana. Esta evaluación se llevó a cabo mediante la predicción hecha por dos herramientas bioinformáticas que funcionan con algoritmos diferentes. Para la identificación, se tomó la secuencia FASTA de cada proteína y se fragmentó para obtener péptidos de 15 aminoácidos, de tal manera que el primer péptido iba de la posición 1 a 15, el segundo péptido de la posición 2 a 16, y así sucesivamente.

De la proteína GP63 se obtuvieron 575 secuencias; de la HSP70, 640, y de la PEPCK, 584, para un total de 1799 secuencias. Todas estas secuencias fueron evaluadas frente a 10 alelos de HLA-DRß*04 por medio de las herramientas NetMHCIIpan y MixMHC2pred, para un total de 39 578 predicciones.

El punto de corte seleccionado para ambas herramientas en este estudio fue %Rank ≤ 1, que quiere decir que, entre más pequeño su valor, mayor afinidad de unión del péptido que se está estudiando, en comparación con otras secuencias al azar. Las secuencias que mostraron una unión fuerte por varios alelos simultáneamente en las dos herramientas empleadas fueron seleccionadas como potenciales epítopos T. Se seleccionaron seis secuencias: tres de la proteína GP63, una secuencia de HSP70 y dos secuencias de PEPCK (cuadro 1).

**Cuadro 1:** Secuencias con unión fuerte a diversos alelos de HLA-DRß*04

#	Código	Secuencia	Alelo de HLA-DRß con unión fuerte (Rank ≤ 1%)		Analogía con especies de *Leishmania* (%)	Analogía con proteína humana (%)
			NETMHCIIpan	MixMHC2pred		
1	GP63^195-210^	ADF**VLYVASVPS**EPG	0402, 0403, 0404, 0405, 0406, 0407, 0408, 0410, 0411, 0417	0401, 0402, 0403, 0404, 0405, 0406, 0407, 0408, 0410, 0411	100	53
2	GP63^267-282^	AGIV**KSVTNLRG**KPF	0401, 0402, 0403, 0404, 0405, 0406, 0407, 0408, 0410, 0411	0401, 0402, 0403, 0404, 0405, 0406, 0407, 0408, 0410, 0411	93,3	20
3	GP63^282-297^	AAPV**INSSTVVA**KAR	0401, 0402, 0404	0401, 0402, 0403, 0404, 0406, 0407, 0411.	93,3	26,6
4	HSP70^169-184^	EVL**RIINEPTAA**AIA	0401, 0402, 0403, 0404, 0406, 0407, 0408, 0410	0401, 0402, 0403, 0404, 0405, 0406, 0407, 0408, 0410, 0411	100	93
5	PEPCK^251-266^	ATPY**RIITDNPT**HAY	0401, 0402, 0403, 0404, 0405, 0406, 0407, 0408, 0410, 0411, 0417	0401, 0404, 0407, 0408	100	NS
6	PEPCK535-550	DVV**IIGEGTDAA**AAI	0401, 0403, 0404, 0406, 0407, 0408.	0401, 0402, 0403, 0404, 0406, 0407, 0408	100	NS

NS: sin analogía significativa; Rank: puntuación de unión HLA-DR-péptido (Rank ≤1 unión fuerte)

La segunda fase consistió en la comparación de la secuencia de estos péptidos con su proteína homóloga frente a otras especies de *Leishmania* causantes de leishmaniasis cutánea americana. La analogía de las secuencias estuvo entre el 93 y el 100 %, lo cual indica que estas regiones están muy conservadas entre las especies y que estos epítopos podrían generar protección cruzada entre especies (figuras 1-3 y cuadro 1).

**Figura 1:**
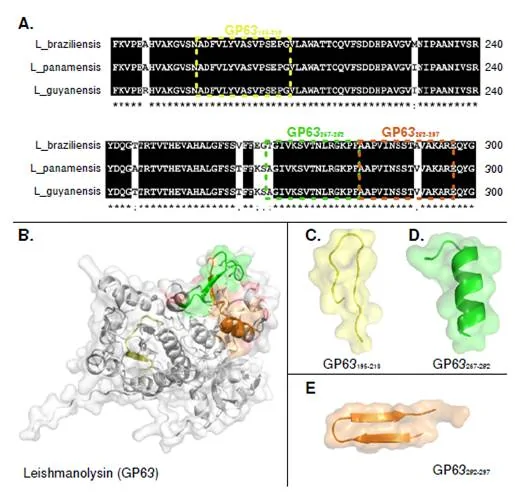
Alineamiento de proteína GP63 entre especies de *Leishmania* y ubicación de péptidos en estudio. **A)** Alineamiento múltiple entre GP63 de *L. braziliensis*, *L. panamensis* y *L. guyanensis*. En los recuadros de línea punteada se encuentra la ubicación de los péptidos en estudio. **B)** Estructura 2D de la proteína GP63 señalando la ubicación de los péptidos. **C, D** y **E.** modelo 3D de los péptidos GP63_195-210_, GP63_267-282_ y GP63_282-297_, respectivamente.

* Analogía entre las secuencias

: Cambio de un aminoácido por otro con características fisicoquímicas similares

. Cambio por un aminoácido con características fisicoquímicas diferentes

**Figura 2:**
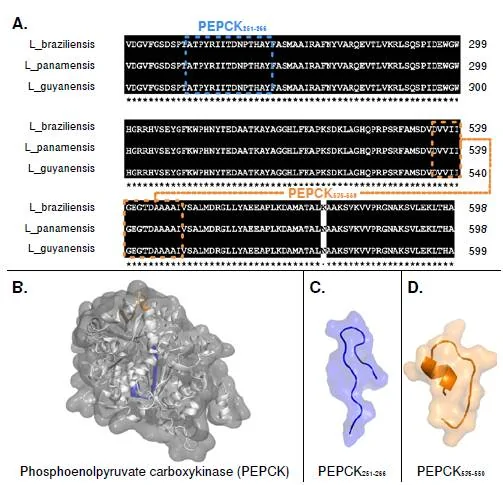
Alineamiento de proteína PEPCK entre especies de *Leishmania* y ubicación de péptidos en estudio. **A)** Alineamiento múltiple entre PEPCK de *L. braziliensis*, *L panamensis* y *L. guyanensis*. En los recuadros de línea punteada se encuentra la ubicación de los péptidos en estudio. **B)** Estructura 2D de la proteína PEPCK señalando la ubicación de los péptidos. **C** y **D.** Modelo 3D de los péptidos PEPCK_251-266_, y PEPCK_535-550_, respectivamente.

* Analogía entre las secuencias

: Cambio de un aminoácido por otro con características fisicoquímicas similares

. Cambio por un aminoácido con características fisicoquímicas diferentes

**Figura 3:**
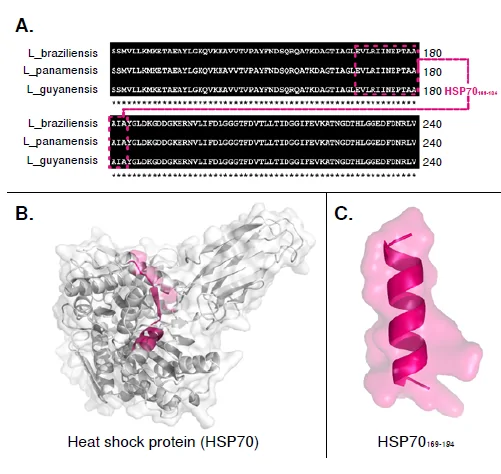
Alineamiento de proteína HSP70 entre especies de Leishmania y ubicación de péptidos en estudio. **A)** Alineamiento múltiple entre HSP70 de L. braziliensis, L panamensis y L. guyanensis. En los recuadros de línea punteada se encuentra la ubicación de los péptidos en estudio. **B)** Estructura 2D de la proteína HSP70 señalando la ubicación de los péptidos. **C)** Modelo 3D del péptido HSP70_169-184_.

* Analogía entre las secuencias

: Cambio de un aminoácido por otro con características fisicoquímicas similares

. Cambio por un aminoácido con características fisicoquímicas diferentes

En la tercera fase, cada secuencia se comparó frente a proteínas humanas para descartar tolerancia o riesgo de inducción de autoinmunidad, en donde la analogía de cada péptido con proteínas humanas estuvo entre el 0 y el 93 %, resaltando que aquellas secuencias que superan el 50 % de similitud no continuaban como candidatos vacunales. Como resultado de este tamizaje, cuatro secuencias cumplieron con las condiciones planteadas: GP63_267-282_, GP63_282-297_, PEPCK_251-266_ y PEPCK_535-550_.

Posteriormente, se procedió a realizar el acoplamiento molecular de cada una de estas secuencias frente al bolsillo de la proteína HLA-DRß 04:01 para validar la unión fuerte de las secuencias a dicho alelo. En el cuadro 2 se anota la energía de interacción entre el receptor y el péptido del mejor modelo seleccionado. Las fuerzas que interactúan entre la proteína y el ligando pueden ser fuerzas favorables -puentes de hidrógeno, interacciones hidrofóbicas y fuerzas electrostáticas- que permiten la unión de las moléculas y posibilitan la estabilidad, mientras que las fuerzas desfavorables producen repulsión entre los aminoácidos. Así, la energía de interacción muestra la suma total de todas las fuerzas de manera que, con menos energía, hay mayor fuerza de unión.

**Cuadro 2:** Energía de interacción por acoplamiento molecular

Péptido	Energía de interacción (kcal/mol)*	Orientación de unión al bolsillo
GP63_267-282_	-9,31	N-terminal a C-terminal
GP63_282-297_	-8,6	N-terminal a C-terminal
PEPCK_251-266_	-9,4	C-terminal a N-terminal
PEPCK_535-550_	-8,71	N-terminal a C-terminal

* Energía de interacción entre secuencias y bolsillo de HLA-DR (kcal/mol)

En el cuadro 2, se señalaa que el péptido PEPCK_251-266_ tiene la menor energía de interacción (expresada como kcal/mol), seguido muy de cerca por GP63_267-282,_ lo cual se correlaciona con una mayor fuerza de unión o estabilidad. Los que mostraron menores energías de interacción fueron PEPCK_535-550_ y GP63_282-297_^.^

En la figura 4, se muestran en detalle los acoplamientos moleculares en en los cuales podemos observar que los residuos ASN62 y ASN69 de la cadena alfa generalmente establecen uniones de tipo puentes de hidrógeno con aminoácidos en el ligando. Por su parte, los residuos TRP61, HIS81, ILE67 e HIS13 de la cadena beta, son importantes para la unión de ligandos en el bolsillo del HLA-DRß*04, lo cual genera uniones favorables, especialmente puentes de hidrógeno.

**Figura 4:**
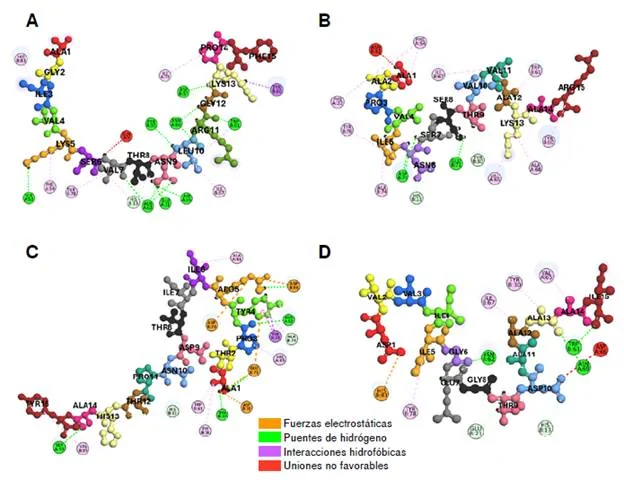
Acoplamiento molecular de bolsillo de HLA-DRβ 04:01 con GP63267-282 (A), GP63282-297 (B), PEPCK251-266 (C) y PEPCK535-550 (D).

Por el lado del péptido, los aminoácidos interactúan principalmente con residuos polares del bolsillo y, aunque se esperaba que la secuencia se ubicara dentro del bolsillo en dirección N-terminal a C-terminal, PEPCK_251-266_ se unió a este en sentido contrario. Los péptidos evaluados presentaron capacidad de unión al bolsillo con diversas interacciones con ambas cadenas y mínimas fuerzas desfavorables o de repulsión, lo que permitiría una unión estable durante el tiempo suficiente para que pueda haber una activación de la respuesta inmunológica.

## Discusión

La selección de epítopos constituye un paso crítico en el diseño de vacunas a partir de subunidades patógenas de parásitos intracelulares como *Leishmania* spp. En este estudio, se priorizaron las proteínas GP63, PEPCK y HSP70 debido a su función biológica, presencia de forma constitutiva e inmunogenicidad.

Por un lado, la metaloproteasa GP63 se encarga de la degradación de las proteínas de la matriz extracelular y de la inhibición del sistema del complemento, que permite el ingreso del promastigote al macrófago ^24^. Por otro lado, la proteína PEPCK hace parte del metabolismo energético del amastigote intracelular, el cual le permite sobrevivir en los ambientes hostiles del fagolisosoma ^25^. La HSP70 juega un rol como chaperona molecular, conservada, y ha demostrado actuar como adyuvante en la activación de las células presentadoras de antígeno por medio de *toll-like receptors* (TLR) ^26^^,^^27^.

Considerando que el control de la leishmaniasis requiere estrictamente una respuesta de tipo Th1 -mediada por la activación de macrófagos, producción de interferones y liberación de óxido nítrico ^28^-, nuestra estrategia se centró exclusivamente en el tamizaje de secuencias con afinidad por CHM-II. Se omitió la búsqueda de epítopos para las células B, dado que la respuesta humoral se ha correlacionado con la progresión de la enfermedad y el fracaso terapéutico ^29^. Este enfoque garantiza que los candidatos seleccionados tienen el potencial de orquestar la inmunidad celular necesaria para eliminar el parásito.

La relevancia de esta estrategia radica en la aproximación de la búsqueda de tipo embudo de selección multipaso que incrementa progresivamente la probabilidad de éxito profiláctico. Al integrar filtros de conservación entre especies y de seguridad (ausencia de homología humana), se garantiza que, desde las fases tempranas, se disminuya el riesgo de tolerancia inmunológica o de inducción de autoinmunidad. Este flujo de trabajo no es exclusivo para *Leishmania* spp.; representa una plataforma adaptable para el diseño de vacunas contra otros agentes patógenos que puedan ser controlados por una respuesta celular fuerte ^25^^,^^30^^-^^33^.

El tamizaje planteado en este estudio permite que, paso a paso, se reduzca el número de secuencias candidatas, a la vez que va aumentando la probabilidad de éxito de las secuencias, tanto en la capacidad de inducción de la respuesta inmunitaria para una amplia gama de especies infecciosas, como en la seguridad. Es indispensable que, desde las fases preclínicas iniciales del desarrollo de productos farmacéuticos basados en proteínas, no haya analogía con las proteínas humanas pues generaría tolerancia inmunológica o el riesgo de inducción de una condición autoinmunológica. Esta estrategia podría ser empleada con diversas proteínas y poblaciones, y frente a distintos agentes infecciosos que requieren una respuesta inmunitaria celular similar a aquella contra *Leishmania* spp., por ejemplo, aquellos agentes patógenos que usan al macrófago como su célula blanco ^34^^-^^37^.

Para refinar esta selección, el acoplamiento molecular *(docking)* permite hacer un análisis de solidez a partir de la resolución estructural. Este análisis se expresa mediante dos variables: la energía libre (kj/mol) y la estabilidad de los complejos péptido-proteína. En este sentido, se revisaron las interacciones dentro de los bolsillos de unión (P1, P4, P6 y P9), debido a que determinan la vida media del complejo en el bolsillo CMH-II y, por lo tanto, la avidez y señalización hacia el receptor de las células T ^34^^,^^35^^,^^38^. Como resultado, la selección de candidatos después del acoplamiento molecular se hace más fina y precisa.

Sin embargo, en las bases de datos hay una escasa disponibilidad de moléculas del CMH humano generadas por rayos X o resonancia magnética *(Magnetic Resonance),* lo cual limita los resultados a pocas isoformas disponibles. Prueba de esto puede encontrarse en otras investigaciones que han tomado el enfoque de predicción para la validación de distintos constructos *in silico* propuestos como vacunas partiendo de las mismas proteínas empleadas en este estudio. La diferencia radica en las distintas isoformas utilizadas del CMH, que incluyen diferentes HLADR -moléculas de CMH humano de clase I e incluso homólogos en ratones-, obteniendo péptidos con secuencias distintas ^36^^,^^37^. Esto no resulta extraño dada la naturaleza de las interacciones inmunológicas.

Por lo anterior, esta estrategia de tamizaje permite un acercamiento a las secuencias que podrían ser candidatas a vacunas y generar una respuesta inmunológica adecuada. Sin embargo, es necesario validar dicha utilidad con experimentación *in vitro* o *ex vivo,* usando directamente células humanas.

En conclusión, a partir de las secuencias de las proteínas GP63, HSP70 y PEPCK, se obtuvieron 1796 secuencias lineales de 15 aminoácidos a lo largo de la longitud total de las mismas. Después del primer filtro y de predicciones bioinformáticas, obtuvimos seis secuencias que cumplieron los criterios de selección, de las cuales solo cuatro pasaron el segundo y el tercer filtro de homología y seguridad.

Finalmente, después del análisis por acoplamiento molecular podemos concluir que GP63_267-282_, GP63_282-297_ y PEPCK_535-550_ son secuencias que tienen una gran probabilidad de ser epítopos naturales de *Leishmania* spp. y desencadenar respuestas en los linfocitos T CD4+ que puedan llevar al control más eficiente de la replicación del parásito por parte del macrófago y, de esta manera, convertirse en alternativas de profilaxis o tratamiento inmunomodulador frente a la leishmaniasis. Estas tres secuencias son valiosas para la comprobar su utilidad en experimentos *in vitro.*

## References

[1] World Health Organization Leishmaniasis.

[2] Instituto Nacional de Salud Sistema de Vigilancia SIVIGILA.

[3] Costa-Da-Silva AC, Nascimento D de O, Ferreira JRM, Guimarães-Pinto K, Freire-De-lima L, Morrot A (2022). Immune responses in leishmaniasis: An overview. Trop Med Infect Dis.

[4] Scott P (2020). Long-lived skin-resident memory T cells contribute to concomitant immunity in cutaneous leishmaniasis. Cold Spring Harb Perspect Biol.

[5] Kedzierski L (2010). Leishmaniasis vaccine: Where are we today?. J Glob Infect Dis.

[6] Liao WW, Arthur JW (2011). Predicting peptide binding to major histocompatibility complex molecules. Autoimmun Rev.

[7] Nagill R, Kaur S (2011). Vaccine candidates for leishmaniasis: A review. Int Immunopharmacol.

[8] Zhang J, He J, Li J, Zhou Q, Chen H, Zheng Z (2020). The immunogenicity and protective immunity of multi-epitopes DNA prime-protein boost vaccines encoding Amastin-Kmp-11, Kmp11-Gp63 and Amastin-Gp63 against visceral leishmaniasis. PLoS One.

[9] Bhowmick S, Ravindran R, Ali N (2008). Gp63 in stable cationic liposomes confers sustained vaccine immunity to susceptible BALB/c mice infected with Leishmania donovani.. Infect Immun.

[10] Mazumder S, Maji M, Das A, Ali N (2011). Potency, efficacy and durability of DNA/DNA, DNA/protein and protein/protein based vaccination using gp63 against Leishmania donovani in BALB/c mice. PLoS One.

[11] Yadav S, Anand A, Goyal N (2023). Challenges and solutions against visceral leishmaniasis.

[12] Rasouli M, Karimi MH, Kalani M, Ebrahimnezhad S, Namayandeh M, Moravej A (2014). Immunostimulatory effects of Leishmania infantum HSP70 recombinant protein on dendritic cells in vitro and in vivo. Immunotherapy.

[13] Zayats R, Mou Z, Yazdanpanah A, Gupta G, Lopez P, Nayar D (2023). Antigen recognition reinforces regulatory T cell mediated Leishmania major persistence. Nat Commun.

[14] Mou Z, Li J, Boussoffara T, Kishi H, Hamana H, Ezzati P (2015). Identification of broadly conserved cross-species protective Leishmania antigen and its responding CD4+ T cells. Sci Transl Med.

[15] Edgar RC (2004). MUSCLE: Multiple sequence alignment with high accuracy and high throughput. Nucleic Acids Res.

[16] Jumper J, Evans R, Pritzel A, Green T, Figurnov M, Ronneberger O (2021). Highly accurate protein structure prediction with AlphaFold. Nature.

[17] Varadi M, Bertoni D, Magana P, Paramval U, Pidruchna I, Radhakrishnan M (2024). AlphaFold Protein Structure Database in 2024: Providing structure coverage for over 214 million protein sequences. Nucleic Acids Res.

[18] PyMOL PyMOL by Schrödinger.

[19] Reynisson B, Barra C, Kaabinejadian S, Hildebrand WH, Peters B, Nielsen M (2020). Improved prediction of MHC II antigen presentation through integration and motif deconvolution of mass spectrometry MHC eluted ligand data. J Proteome Res.

[20] Racle J, Guillaume P, Schmidt J, Michaux J, Larabi A, Lau K (2023). Machine learning predictions of MHC-II specificities reveal alternative binding mode of class II epitopes. Immunity.

[21] Gonzalez-Galarza FF, McCabe A, Dos Santos EJM, Jones J, Takeshita L, Ortega- Rivera ND (2020). Allele frequency net database (AFND) 2020 update: Gold-standard data classification, open access genotype data and new query tools. Nucleic Acids Res.

[22] The Allele Frequency Net Database Allele, haplotype and genotype frequencies in worldwide populations.

[23] Xu X, Yan C, Zou X (2018). MDockPeP: An ab-initio protein-peptide docking server. J Comput Chem.

[24] Chan A, Ayala JM, Alvarez F, Piccirillo C, Dong G, Langlais D (2021). The role of Leishmania GP63 in the modulation of innate inflammatory response to Leishmania major infection. PLoS One.

[25] Barazandeh AF, Mou Z, Ikeogu N, Mejia EM, Edechi CA, Zhang WW (2021). The phosphoenolpyruvate carboxykinase is a key metabolic enzyme and critical virulence factor of Leishmania major. J Immunol.

[26] Rico AI, Gironès N, Fresno M, Alonso C, Requena JM (2002). The heat shock proteins, Hsp70 and Hsp83, of Leishmania infantum are mitogens for mouse B cells. Cell Stress Chaperones.

[27] Prasanna P, Upadhyay A (2021). Heat shock proteins as the druggable targets in leishmaniasis: Promises and perils. Infect Immun.

[28] Paton H, Sarkar P, Gurung P (2025). An overview of host immune responses against Leishmania spp. infections. Hum Mol Genet.

[29] Sacks D, Noben-Trauth N (2002). The immunology of susceptibility and resistance to Leishmania major in mice. Nat Rev Immunol.

[30] Jamal F Shivam P, Kumari S Singh MK, Sardar AH Pushpanjali (2017). Identification of Leishmania donovani antigen in circulating immune complexes of visceral leishmaniasis subjects for diagnosis. PLoS One.

[31] Mou Z, Li J, Boussoffara T, Kishi H, Hamana H, Ezzati P (2015). Identification of broadly conserved cross-species protective Leishmania antigen and its responding CD4+ T cells. Sci Transl Med.

[32] Elmahallawy EK, Alkhaldi AAM, Saleh AA (2021). Host immune response against leishmaniasis and parasite persistence strategies: A review and assessment of recent research. Biomed Pharmacother.

[33] Ikeogu NM, Akaluka GN, Edechi CA, Salako ES, Onyilagha C, Barazandeh AF (2020). Leishmania immunity: Advancing immunotherapy and vaccine development. Microorganisms.

[34] McFarland BJ, Beeson C (2002). Binding interactions between peptides and proteins of the class II major histocompatibility complex. Med Res Rev.

[35] Ferrante A (2013). For many but not for all: How the conformational flexibility of the peptide/MHCII complex shapes epitope selection. Immunol Res.

[36] Ferreira-Sena EP, Hardoim D de J, Cardoso F de O, d’Escoffier LN, Soares IF, Carvalho JPR da S (2023). A new strategy for mapping epitopes of LACK and PEPCK proteins of Leishmania amazonensis specific for major histocompatibility complex class I. Int J Mol Sci.

[37] Saha S, Vashishtha S, Kundu B, Ghosh M (2022). In-silico design of an immunoinformatics based multi-epitope vaccine against Leishmania donovani. BMC Bioinformatics.

[38] Yang Y, Wei Z, Cia G, Song X, Pucci F, Rooman M (2024). MHCII-peptide presentation: an assessment of the state-of-the-art prediction methods. Front Immunol.

